# Isolation and Identification of Porcine Epidemic Diarrhea Virus and Its Effect on Host Natural Immune Response

**DOI:** 10.3389/fmicb.2019.02272

**Published:** 2019-10-04

**Authors:** Shaoju Qian, Weida Zhang, Xiangchao Jia, Zhijian Sun, Yang Zhang, Yuncai Xiao, Zili Li

**Affiliations:** ^1^State Key Laboratory of Agricultural Microbiology, Department of Preventive Veterinary Medicine, College of Veterinary Medicine, Huazhong Agricultural University, Wuhan, China; ^2^Key Laboratory of Preventive Veterinary Medicine in Hubei Province, The Cooperative Innovation Center for Sustainable Pig Production, Wuhan, China; ^3^Key Laboratory of Development of Veterinary Diagnostic Products, Ministry of Agriculture of the People’s Republic of China, Wuhan, China

**Keywords:** porcine epidemic diarrhea virus, neonatal fc receptor, Toll-like receptor, NF-κB, neutralizing antibody

## Abstract

Porcine epidemic diarrhea (PED) is a highly infectious intestinal disease caused by porcine epidemic diarrhea virus (PEDV). A PEDV strain was isolated from the piglet intestinal tract in Vero cells in Jiangsu Province, designated as the JS-A strain. PEDV was identified as the isolated virus by cytopathology, immunofluorescence assay, western blotting, transmission electron microscopy, and sequence analysis. The full-length genome of the JS-A isolate and the S gene were systematically analyzed, indicating that PEDV JS-A belongs to the G2a subtype, which is closely related to the prevalent PEDV in many countries and different from many current vaccines. Animal regression tests showed that piglets that are orally infected with the virus continue to develop diarrhea with yellowish and unpleasant odors. Further, piglets showed reduced food consumption and weight loss in the challenged group, while there were no abnormalities in the control group. In addition, Toll-like receptors (TLRs), RIG-I, and the downstream medium gene in the intestinal mucosa of newborn pigs infected with PEDV JS-A strain were studied. The neonatal Fc receptor (FcRn) was the only IgG transport receptor and protected IgG from degradation. Therefore, PEDV JS-A infection might inhibit FcRn expression by down-regulating TLRs and downstream signaling molecules. Taken together, isolation of the JS-A variant contributes to evolutionary analysis of the diarrhea virus. Further, the experimental infection model lays a foundation for further research related to vaccine development and the antiviral natural immune response of infected piglets, which helps us to better understand PEDV pathogenesis and immune mechanism.

## Introduction

Porcine epidemic diarrhea virus (PEDV) causes severe enteritis, watery diarrhea, vomiting, dehydration, and even death in suckling piglets. In the 1970s, the first PEDV outbreak occurred in Europe (Wood, 1977). Subsequently, the virus affected many countries such as the Czech Republic, Belgium, Hungary, South Korea, Italy, and Thailand (Song and Park, 2012). Since 2010, a large-scale outbreak of PEDV has occurred in swine herds around China. Then, the emergence of PEDV occurred in Ohio in the United States in 2013, which spread throughout the United States (Huang et al., 2013). Currently, due to lack of effective vaccines, isolation and identification of new PEDV strains to develop appropriate vaccines for prevention and control of the disease have garnered great interest.

PEDV belongs to the genus Alphacoronavirus within the Coronaviridae family (Brian and Baric, 2005). Its genome size is about 28 kb, and it possesses at least seven open reading frames (ORF): ORF1a, ORF1b, spike (S), ORF3, envelope (E), membrane (M), and nucleocapsid (N). PEDV S protein plays a critical role in inducing neutralization antibodies, specific receptor binding, and membrane fusion. In addition, the S gene has shown a high degree of genetic variability. Therefore, it is often used to analyze PEDV genetic variation. According to phylogenetic analysis of S genes, PEDV strains have been divided into two clusters: genome I subtype and genome II subtype (Lee et al., 2010; Li et al., 2013; Chen et al., 2014). Further, several variant PEDV strains have been identified, which were characterized by insertion and deletion (INDEL) in labeled (S) genes named S-INDEL PEDV. The pathogenesis of PEDV is strain specific. Studies on its pathogenesis in piglets suggest that the PEDV non-S-INDEL strain has stronger pathogenicity compared to the PEDV S-INDEL strain (Chen et al., 2014; Yamamoto et al., 2015; Wang et al., 2016). PEDV JS-A belongs to the non-S-INDEL strain.

Upon viral infection, the host innate immune response is the first line of defense (Moynagh, 2005). NF-κB regulates expression of numerous components of the immune system, which is thought to be the hallmark of most infection, including coronaviruses (DeDiego et al., 2014). RNA viruses interact with pattern-recognition receptors (PRRs), including Toll-like receptors (TLRs) and RIG-I-like receptors (RLRs). Several *in vitro* studies have been conducted to clarify the role of PEDV in innate immune responses at the cellular level. Several other studies have determined that PEDV nucleocapsid protein (N) inhibits the production of type I and III interferons (IFNs) (Ding et al., 2014; Cao et al., 2015a; Shan et al., 2018). PEDV non-S-INDEL infection inhibits the induction of proinflammatory cytokines and IFN-I by down-regulating TLRs and downstream signaling molecules (Temeeyasen et al., 2018).

Neonatal Fc receptor (FcRn) has been fully characterized to transfer maternal IgG to the fetus or newborn and protect IgG from degradation. In addition to IgG, FcRn binds to albumin, which regulates liver damage (Pyzik et al., 2017). Transmissible gastroenteritis virus (TGEV) infection up-regulates pFcRn expression and activates the NF-κB signaling pathway in IPEC-J2 cells (Guo et al., 2016a). In our study, the pathogenicity of JS-A in 5-day-old piglets was studied by clinical evaluation, quantitative analysis of virus copy number in the feces, histology, and immunohistochemistry. We also studied the gene regulation of TLRs, RIG-I, and downstream signaling pathways in the intestinal mucosa of pigs newly infected with JS-A. The purpose of this study was to investigate the genomic characteristics and pathogenicity of JS-A and the expression of TLRs, RIG-I, NF-κB, and FcRn in the intestinal mucosa of infected piglets.

## Materials and Methods

### Clinical Samples and Virus Isolation

Fecal samples and intestinal contents from piglets suffering from severe watery diarrhea were collected in a pig farm from Jiangsu Province. PEDV-positive samples were detected by real-time polymerase chain reaction (RT-PCR), based on the N gene, as previously described (Lee et al., 2015). Clinical samples were homogenized in DMEM (HyClone, Logan, UT, USA), centrifuged at 4,000 rpm/min, and filtered through a 0.22-μm filter (Millipore, MA).

Vero (ATCC, CCL-81) cells were obtained from the China Center for Type Culture Collection (Wuhan, China) and were cultured in DMEM containing 10% fetal bovine serum (FBS, Gibco, Waltham, MA, USA). Vero cells were incubated with 1 mL of filtered inoculum for 2 h. After virus adsorption, cells were maintained in DMEM containing 8 μg/mL trypsin (Gibco, USA) and antibiotics. After 2 h, the cells were maintained in DMEM containing 8 μg/mL trypsin at 37°C in 5% CO_2_ until a cytopathic effect became visible. The supernatant was collected after the cytopathic effect was observed. The virus was cloned and purified by plaque purification three times and tested by TCID_50_.

### Immunofluorescence Assay

At 24 h post-infection, cells were fixed with 4% paraformaldehyde in PBS for 15 min and permeabilized with 0.1% Triton X-100 for 10 min. Subsequently, cells were incubated with PBST containing 5% skim milk for 1 h, followed by primary (PEDV polyclonal antibody; prepared in our laboratory) and secondary [fluorescein isothiocyanate (FITC)-labeled goat anti-rabbit IgG; ABclonal, China] antibodies for 1 h. Finally, the samples were stained with DAPI for 15 min and examined using a fluorescence microscope (Olympus IX73, Japan).

### Western Blot

At 24 h post-infection, cells lysates were prepared for 12% SDS-PAGE, and proteins were electroblotted onto a polyvinylidene difluoride membrane (Bio-Rad, USA). PEDV-N polyclonal antibody (prepared in our laboratory) was used as a primary antibody, followed by HRP-conjugated goat anti-rabbit IgG or anti-mouse IgG (ABclonal, China) as secondary antibodies. Proteins were visualized as described previously (Guo et al., 2016b).

### Electron Microscopy

PEDV-infected Vero cells were examined by electron microscopy (EM), following previously described procedures (Chen et al., 2014). These samples were negatively stained with 2% sodium phosphotungstic acid (pH 6.8) and examined using a Hitachi Model H-7650 TEM (Hitachi H-7000FA, Japan).

### Phylogenetic Analysis

Total RNAs were extracted using TRIzol^@^ reagent (Invitrogen, Carlsbad, CA, USA), and total RNA was reverse transcribed into cDNA using reverse transcriptase (TaKaRa, Osaka, Japan). The whole genome of JS-A was sequenced using 25 pairs of primers previously reported in Table 1 by Chen (Chen et al., 2012). Phylogenetic trees with 25 strains (Table 2) full-length genome and S-nucleotide sequences were generated using the distance-based adjacency method in MEGA7.

**Table 1:** Primers for amplifications of the PEDV genomic fragments by RT-PCR.

Name	Sequence (5′ to 3′)	Position in PEDV genome	Size (bp)
PP1F	GTGGAATTTCATTAGGTTTG	123-142	1,380
PP1R	AAGCTTACGTATGAACCAAG	1502-1483	
PP2F	TGCTGGTCATGTTGTTGTTG	1421-1440	1,488
PP2R	TAGAAAGCGAAGCCATCAAC	2908-2889	
PP3F	ATTGAAACTTCTTTTGTGGA	2817-2836	1,451
PP3R	TTCTCATTTGCAGCATTAAC	4267-4248	
PP4F	CTGCTCTCTCCTTGGATTCT	3940-3959	1,478
PP4R	AGTATGGTCTAGCATGTGGA	5417-5398	
PP5F	TGTCACAGACAACAAACCTG	5300-5319	1,487
PP5R	CATCACCAAAGACATCAAAA	6786-6767	
PP6F	ATTGGTAATGTGATGCCTTT	6609-6628	1,323
PP6R	AAAGCTTAGTGCAAAGAAAG	7931-7912	
PP7F	TTTCAAAGGTTAAGAAATTCT	7861-7881	1,408
PP7R	CTCACAGTGGGTGGTGTGTAT	9268-9248	
PP8F	CGTTATACACACCACCCACT	9244-9263	1,304
PP8R	GGTAACAACAAAGCACACAA	10547-10528	
PP9F	CAGAGCATTTTGATTACCAT	10465-10484	1,470
PP9R	CAACTATGCCATCTCCTTCT	11934-11915	
PP10F	GGTGTGAGCGTATTGTTAAG	11842-11861	1,266
PP10R	AATGCATAGACACGATGAAT	13107-13088	
PP11F	TTTGATTAAGGTAGGTGCTT	13012-13031	1,397
PP11R	CAACACCATCAATAAGGTTC	14408-14389	
PP12F	TATGGTGGTTGGGACAATAT	14363-14382	946
PP12R	CTTCCAAAAGTGTGACAGAA	15308-15289	
PP13F	ATTGCTTGAACGTTATGTGT	15142-15161	1,676
PP13R	CATTACCCTTGCAAAAGATC	16817-16798	
PP14F	TTAAGCCTGATGTCTTCTTG	16674-16693	1,691
PP14R	CCTACAGCGAGTATCAAAAC	18364-18345	
PP15F	TGGACATGTATCCAGAATTT	18312-18331	1,455
PP15R	GCATGGAATAACCACACTTC	19766-19747	
PP16F	AAAATGTGGAGGTGGATGTT	19670-19689	1,362
PP16R	GGCCCAATGTTTTATTATCG	21031-21012	
S-1F	TAAGTTGCTAGTGCGTAAT	20572-20590	1,692
S-1R	GTCAACACAGAAAGAACTA	22263-22245	
S-2F	TGAGTCATGAACAGCCAA	22003-22020	1,511
S-2R	CTGGTTGCGCTGTAGAA	23513-23497	
S-3F	GCGTCTCTCATAGGTGGT	23491-23508	1,310
S-3R	GTCCAAGAAACATCACTG	24801-24784	
ORF3·EF	CGTGCAGTGATGTTTCTTGGAC	24780-24801	895
ORF3·ER	TTATACGTCAATAACAGTACT	25674-25654	
EMF	CTTCACTTGTCACCGGTTGT	25557-25576	1,097
EMR	CCTCAGTACGAGTCCTATAAC	26549-26529	
MF	GGCGAATTCAATATGTCTAACGGTTC	25670-25695	675
MR	CGCGTCGACCCATAAAGTTTCTGTT	26368-26362	
NF	GGATCCATGGCTTCTGTCAGCTTT	26107-26218	1,332
NR	GACGTCACATTGTTTAATTTCCTGTACC		
3F	GGAAAAGAAGAACAAGCGT	27355-27373	580
3R	ATCGGTCACCAGTATTTTTTTTTTTTTTT		

**Table 2:** Comparison of nucleotide sequences of the JS-A isolate with other strains.

Strains	Countries	Accession	Strains	Countries	Accession
JS-A	China	MH748550	MEX/124/2014	USA	KJ645700
LZC	China	EF185992	MN	USA	KF468752
AJ1102	China	JX188454	PC21A	USA	KR078299
AH2012	China	KC210145	USA/Colorado/2013	USA	KF272920
GD-B	China	JX088695	CH/JX-2/2013	China	KJ526096
CH-S	China	JN547228	CH/JX-1/2013	China	KF760557
IA2	USA	KF468754	CH/HNAY/2015	China	KR809885
SM98	Korea	GU937797	LC	China	JX489155
Attenuated DR13	Korea	JQ023162	FL2013	China	KP765609
CV777	Switzerland	AF353511	DR13	Korea	JQ023162
YN144	China	KT021232	JS2008	China	KC109141
JS-HZ2012	China	KC210147	Belgorod/dom/2008	Russia	MF577027
GD-1	China	JX647847			

### Inoculation of Piglets With Porcine Epidemic Diarrhea Virus Strain JS-A

This study was implemented according to the Guide of the Scientific Ethics Committee of Huazhong Agricultural University (HZAUSW-2018-011). Twelve 5-day-old piglets were purchased from a commercial pig farm, both PEDV RNA and antibody negative, and were randomly divided into two groups (6 piglets/group) in two separate rooms. Piglets were fed a mixture of liquid milk replacer and had free access to water. After 1 day of adaptation, piglets in the challenged group received 3 × 10^5^ TCID_50_ PEDV JS-A orally, while piglets in the control group received 3 mL maintenance medium orally. The piglets were observed daily for signs of vomiting, diarrhea, lethargy, weight, and body temperature until the end of the experiment. Intestinal segments, including the jejunum and ileum, were collected after euthanasia of three of six piglets in each group, selected at random, at 4 dpi for histopathological and immunohistochemical examinations using the method described previously (Lee et al., 2010). At the end of the experiment (21 dpi), the remaining piglets were euthanized. Expressions of mRNA for TLRs, NF-κB and FcRn on piglet intestinal mucosa in each group were detected by RT-qPCR at 4 and 21 dpi.

### Real-Time Polymerase Chain Reaction

Viral RNA of PEDV in piglet feces or intestinal mucosa was detected as previously described (Subramaniam et al., 2018). The specific porcine primers are referenced in previous publications in Table 3 (Guo et al., 2016a; Temeeyasen et al., 2018) were synthesized by the GenScript Company (Nanjing, China). The N gene amplified from the PEDV JS-A strain was cloned into pMD18-T (Takara, Dalian). The known plasmid concentrations were serially diluted 10-fold to generate a standard curve in each plate. The amount of PEDV viral RNA in the test sample was calculated based on the cycle curve threshold (Ct) value of the standard curve. Expression levels of genes were calculated relative to the expression of GAPDH using the delta-delta cycles to threshold (2^−ΔΔCt^) method.

**Table 3:** Primers used for RT-qPCR analysis of genes expression of pig intestinal mucosa.

Gene name	Primer sequence (5′−3′)	Amplicon length (bp)	Accession number
GAPDH-F	GGAAAGGCCATCACCATCTT	85	XM_021091114.1
GAPDH-R	CATGGTCGTGAAGACACCAG		
FcRn-F FcRn-R TLR2-F	GGCGACGAGCACCACTACTG AGCCGACCATGATTCCAACC GAGTCTGCCACAACTCAAAGA	88 68	HQ026019.1 XM_005653576.3
TLR2-R TLR3-F	CAGAACTGACAACATGGGTAGAA GCGGTCCTGTTCAGTTTCT	72	KT735340.1
TLR3-R TLR4-F	AAGGCATCTGCTGGGATTT AACTGCAGGTGCTGGATTTAT	74	AB078418.1
TLR4-R TLR7-F	CCGTCAGTATCAAGGTGGAAAG CCCAGGTCCTCGAATCATTAC	77	DQ647699.2
TLR7-R TLR8-F	CATTAAGAGGCAAGGAGGAAGA CTTTGATGATGACGCTGCTTTC	77	KF019635.1
TLR8-R TLR9-F	GGTGTGTCACTCCTGCTATTC CCTCACACATCTCTCACTCAAG	82	KC860785.1
TLR9-R RIG-I-F	GGTGACAATGTGGTTGTAGGA GAGCCCTTGTGGATGCTTTA	91	KC011279.1
RIG-I-R	GGGTCATCCCTATGTTCTGATTC		
TRIF-F	CTCCGGTGCAGTCAAACA	91	KC969185.1
TRIF-R	GGTAGTGTGTGCTGGTTTCT		
MyD88A-F	GGCAGCTGGAACAGACCAA	41	EU056736.1
MyD88A-R	GGCAGGACATCTCGGTCAGA		
MyD88B-F	TGCAGGTGCCCATCAGAAG	64	EU056737.1
MyD88B-R	TGATGAACCGCAGGATGCT		
NF-κB1(p105)-F	GAGGTGCATCTGACGTATTC	118	NM_001048232.1
NF-κB1(p105)-R	CACATCTCCTGTCACTGCAT		
NF-κB1(p50)-F	AAGCACGGAACTGTAGACAC	107	KC316024.1
NF-κB1(p50)-R	TCTGTGGTTTCTGTGACTTTCC		
RELA-F	ACATGGACTTCTCAGCCCTTCTGA	285	CN155798.1
RELA-R	CCGAAGACATCACCCAAAGATGCT		
TRAF6-F	GGGAACGATACGCCTTACAA	156	NM_001105286.1
TRAF6-R	CTCTGTCTTAGGGCGTCC		

### IgG/IgA Enzyme-Linked Immunosorbent Assay and Neutralization Assay

The PEDV-specific IgG/IgA antibodies were measured in serum samples by ELISA as previously described (Subramaniam et al., 2018). Polystyrene 96-well microplates were coated with purified PEDV N diluted in PBS containing Tween-20 (PBST) and incubated overnight at 4°C, followed by the removal of the coating solution. The plates were blocked with 100 μL 5% skimmed milk in PBST and incubated at 37°C for 30min. After which, 100 μL of serum samples (diluted 1:200) per well was added and incubated at 37°C for 1 h, respectively. After washing the plates, HRP-conjugated goat anti-swine IgG or goat anti-swine IgA was incubated for 1 h. The plates were washed and incubated with TMB solution (Sigma-Aldrich) for 10 min. The absorbance was measured at 450 nm with a termination solution (2 M H_2_SO_4_).

PEDV isolate JS-A was used to determine the presence of neutralization (PEDV nucleocapsid protein) in serum samples as previously described (Oh et al., 2014). Vero cells were grown to 90% confluence in the 96-well plates. Further, 200 TCID_50_ of JS-A virus was diluted in DMEM in a 50 μL volume. Subsequently, 50 μL of 2-fold serial dilutions of inactivated serum samples was added to the diluted virus and incubated at 37°C for 1 h. The mixture was inoculated into Vero cells and incubated at 37°C for 1 h. After removing the mixture and washing the plates, the virus in the maintenance medium was incubated in a 37°C and 5% CO_2_ incubator for 2 days. The neutralization titer was calculated as the reciprocal of the highest serum dilution that neutralizes PEDV infection.

### Statistical Analysis

Data were analyzed as mean ± SEM. Differences among groups were performed by one-way ANOVA using GraphPad Prism software. The significance level for all analyses was set as *p < 0.05, **p < 0.01 and ***p < 0.001.

## Results

### Virus Isolation and Characterization

**Figure 1:**
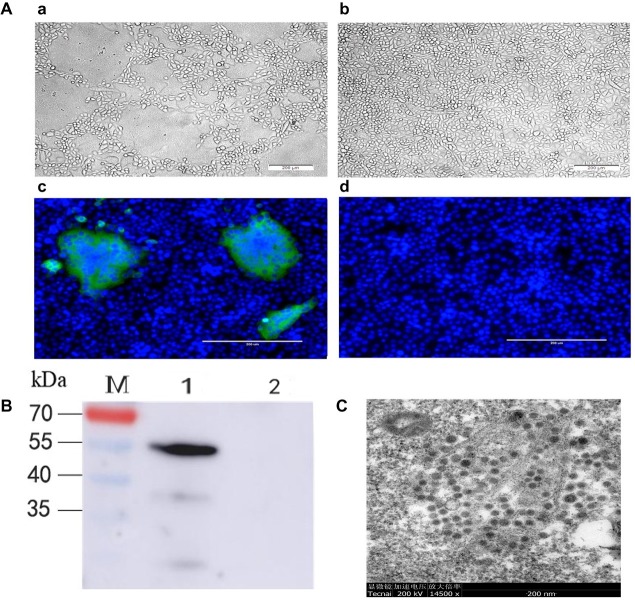
The isolated PEDV strain JS-A was identified. **(A)** Simulated infected Vero cells of 24 hpi **(a)** and negative control Vero cells at 24 hpi **(b)**. PEDV-infected **(c)** or negative control Vero cells **(d)** were examined by IFA at 24 hpi using polyclonal antibodies against PEDV N protein. **(B)** PEDV-infected or negative control Vero cells were subjected to analysis at 24 hpi with polyclonal antibodies against PEDV N protein by western blot; **(C)** electron microscopic images of purified PEDV particles were analyzed.

The treated samples were inoculated onto Vero cells. Cytopathic effects, including cell fusion, syncytium, and detachment, were observed at 36 h post-infection (hpi). No cytopathic effects were observed in the control wells (Figure 1A). To confirm the occurrence of virus multiplication, the infectivity of the JS-A strain (purified by plaque in Vero cells) was assessed by western blot (WB) and IFA using the PEDV N protein polyclonal antibody. IFA results demonstrated a green signal in infected Vero cells, but not in the mock-infected cells (Figure 1A). Western blot analysis also detected N protein in PEDV-infected Vero cells (Figure 1B). According to the structural analysis of the infected cells by transmission electron microscopy, PEDV virus particles were round and showed obvious radial protrusion (Figure 1C), which is typical of coronaviruses. The plaque-purified virus was titrated to 10^5.625^ TCID_50_/mL.

### Phylogenetic Tree Analysis of JS-A Genomic Sequence

**Figure 2:**
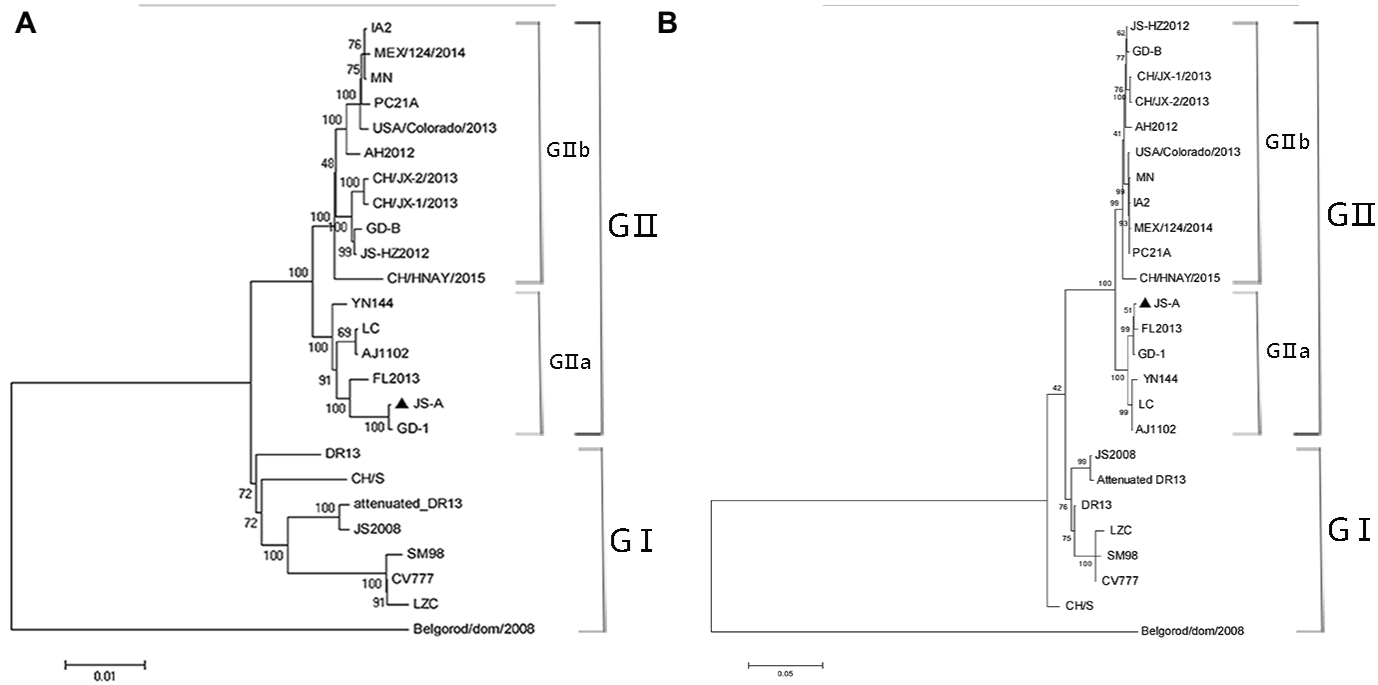
Phylogenetic analysis was performed using the nucleotide sequences of the complete genome **(A)** and S genes **(B)** of the PEDV strain from the GenBank database. JS-A is represented by a triangle. The phylogenetic tree was constructed by the adjacency method in MEGA 7 (http://www.megasoftware.net). Bootstrap analysis was performed using 1,000 iterations and the bootstrap value was indicated on each branch. Scale bar indicates nucleotide substitution at each site.

The JS-A genome was sequenced and deposited in GenBank (MH748550). To further understand the evolutionary relationship between JS-A and other PEDV strains, a phylogenetic tree was constructed based on its genome and the S gene sequence (Figure 2). These two phylogenetic trees all indicated that PEDV was mainly divided into two groups: group I consists mainly of classical strains and vaccine strains, while group II consists mainly of variant strains of China and mutant strains of the United States and South Korea. PEDV JS-A shared homology with the GD-1 strain of 99.9%, belong to non-S INDEL strain. Homology with the classic strain CV777, LZC, and SM98 was 96.8, 96.5, and 96.6%, respectively. The results showed that the S gene had 15 base insertions at 20806–20817 nt and 21,046–21,048 nt, and nine base deletions at 21,114–21,119 nt and 24,225–24,227 nt. It is currently a prevalent species compared to the classical strain CV777.

### Clinical Observations in Pigs Challenged With JS-A

**Figure 3:**
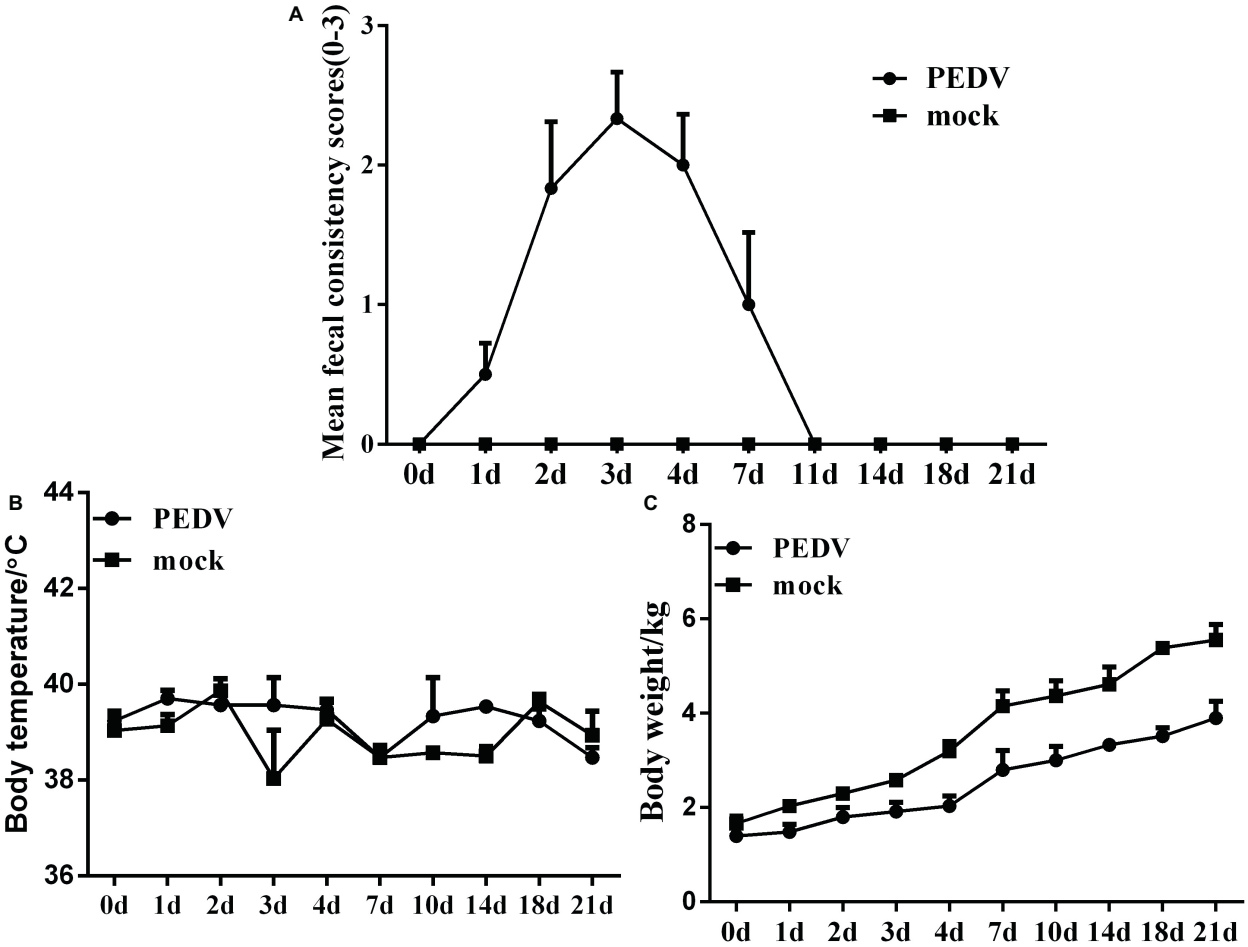
Pathogenicity analysis of PEDV JS-A. **(A)** Diarrhea of piglets in different groups. The severity of diarrhea was scored based on visual examination; 0 = normal and no diarrhea; 1 = mild and fluidic diarrhea; 2 = severe watery diarrhea; with scores of 1 or more considered diarrheic. **(B)** The average body temperature changes in each group. **(C)** The changes in average body weight of each group.

To explore the pathogenicity of PEDV JS-A in piglets, we observed their clinical symptoms. All piglets from the control group were in great health throughout the study without observation of clinical symptoms. However, three of six piglets in the challenged group developed diarrhea at 1 dpi, and the additional three piglets developed various degrees of diarrhea, vomiting, lethargy, and anorexia at 2–6 dpi. Drowsiness, anorexia, and watery diarrhea developed most severely at 3–6 dpi, and piglets gradually recovered thereafter (Figure 3A). No significant temperature change was observed between the two groups (Figure 3B). The infected group showed significant weight loss, while the uninfected piglets experienced weight gain (Figure 3C). Despite watery diarrhea with vomiting, drowsiness, and anorexia, no deaths occurred during the study period. These results indicate that JS-A is pathogenic to newborn pigs.

### Fecal Shedding, Virus Distribution, and Neutralizing Antibody Against JS-A

**Figure 4:**
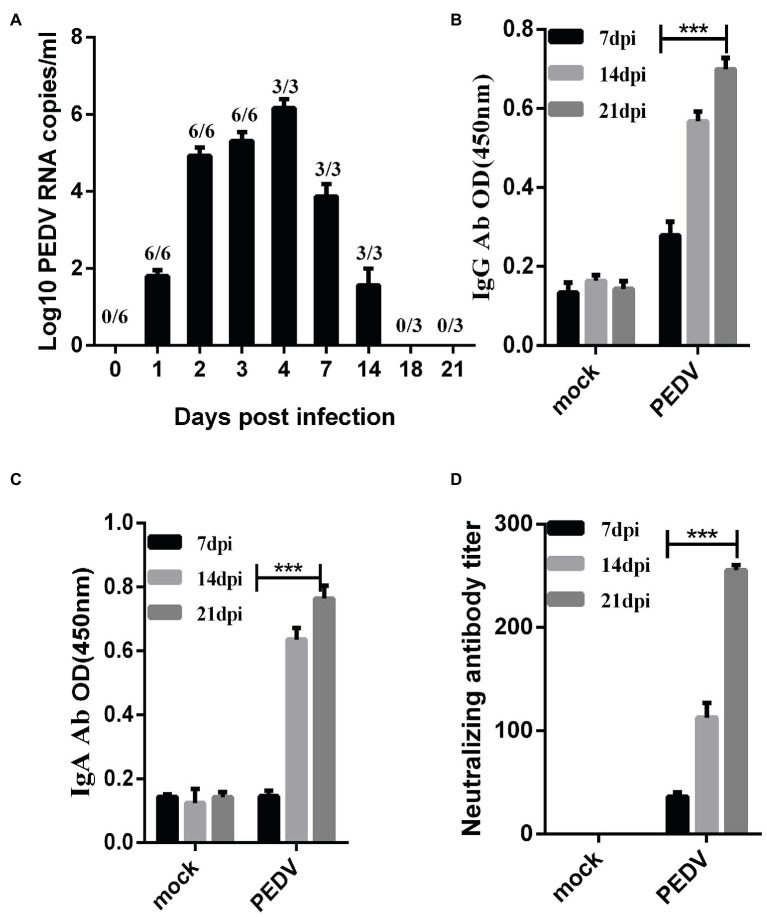
Fecal detoxification and humoral immune response in piglets challenged with PEDV-JS-A. **(A)** Fecal virus shedding in PEDV-challenged piglets. **(B)** Detection of PEDV IgG in serum samples from PEDV-challenged piglets. **(C)** PEDV IgA detection in serum samples from PEDV-challenged piglets. **(D)** Detection of neutralizing antibodies in serum samples of immunized piglets. (Data were analyzed as mean ± SEM) (*n* = 3 for the JS-A group, and statistically significant for the unchallenged group by Student’s t test. ^***^p < 0.001).

To determine the fecal viral shedding, we attempted to detect viral RNA in rectal swab samples from 1 to 21 dpi by RT-PCR. Viral RNA was detected in 6/6 rectal swab samples collected from 1 to 14 dpi in the challenged group. PEDV RNA copies reached the peak of 10^4^–10^7^ copies/mL in the homogenate rectal swab supernatant at 2–7 dpi, and the PEDV RNA was detected up to 14 dpi (Figure 4A). Notably, PEDV RNA was not detected in piglet samples from negative controls throughout the study.

To determine when PEDV IgG, IgA, and neutralizing antibodies were detected in infected piglets, PEDV IgG, IgA, and neutralizing antibodies were measured in serum samples collected at 7, 14, and 21 dpi. The OD values of serum IgG and IgA in piglets inoculated with PEDV gradually increased at 7, 14, and 21 dpi, and the antibody titer gradually increased and peaked at 21 dpi (Figures 4B,C). Neutralizing antibodies in collected serum samples were detected, and it was found that the antibody titer gradually increased and reached a peak at 21 dpi, as high 1:255 (Figure 4D). PEDV IgG, IgA, and neutralizing antibodies were not observed in the serum of control piglets.

### Gross Pathology and Histopathology

**Figure 5:**
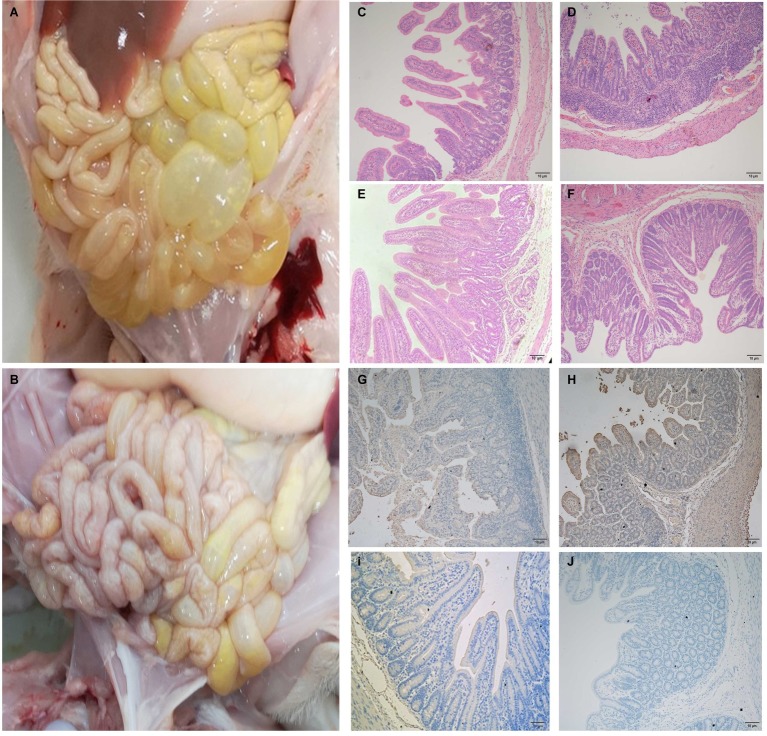
The intestinal microscopic lesions of piglets. **(A)** Macroscopic damage of piglets challenged with PEDV at 4 dpi. **(B)** Macroscopic view of 4 dpi negative control piglets. **(C,D)** H&E stained jejunal **(C)** and ileal **(D)** tissue sections of piglets challenged with PEDV. **(E,F)** H&E stained jejunal **(E)** and ileal **(F)** tissue sections of negative control pigs. **(G,H)** PEDV challenge immunohistochemical staining of jejunal **(G)** and ileal **(H)** tissue sections of piglets. Immunohistochemically stained jejunal **(I)** and ileal **(J)** tissue sections of **(I,J)** negative control pigs.

In order to ascertain changes in piglets infected with JS-A about the overall histology and pathology, three of six piglets in each group were randomly selected for autopsy at 4 dpi. The typical gross lesion seen in infected pigs is the accumulation of large amounts of yellow watery contents in the small intestine, resulting in thin and transparent intestinal wall and gas expansion due to atrophy of intestinal villi (Figure 5A). No other injuries were observed in any organs of the control pigs (Figure 5B). Histopathological examination showed necrosis of small intestinal epithelial cells, atrophy of intestinal villi and vacuolation in infected pigs compared with the control group (Figures 5C,D), but the intestines of the negative control were normal (Figures 5E,F). Consistent with histopathological findings, PEDV antigen was found in the cytoplasm of infected atrophic intestinal villous epithelial cells (Figures 5G,H), and no PEDV IHC antigen was found in uninfected piglets (Figures 5I,J).

### Porcine Epidemic Diarrhea Virus JS-A Infection Suppresses Neonatal Fc Receptor Expression Through Down-Regulation of Toll-Like Receptors and Downstream Signaling Molecules

**Figure 6:**
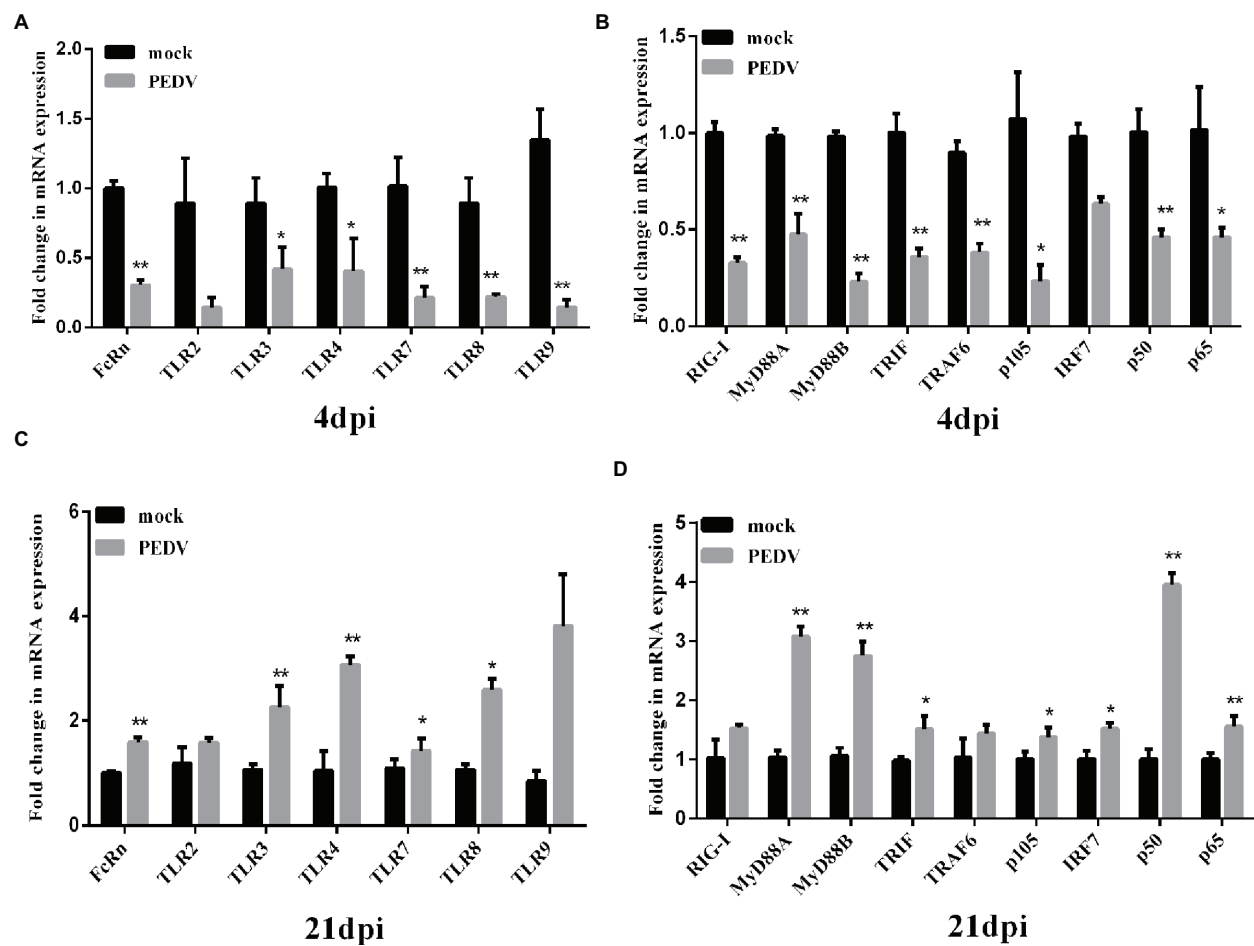
Changes in FcRn, TLRs and its downstream-signaling molecules mRNA expression on intestinal mucosa of piglets induced by PEDV JS-A at 4 and 21 dpi. **(A,C)** The mRNA expressions of FcRn, TLR2, TLR3, TLR4, TLR7, TLR8, TLR9 at the intestinal mucosa from piglets after infection with JS-A at 4dpi **(A)** and 21dpi **(C)** by RT-qPCR. **(B,D)** The mRNA expressions of RIG-I, TRIF, MyD88A, MyD88B, IRF7, TRAF6, NF-kB1 (p105), NF-kB1 (p50), RelA (p65) at the intestinal mucosa from piglets after infection with JS-A at 4dpi **(B)** and 21dpi **(D)** by RT-qPCR. All samples were tested in triplicate and the results are expressed as fold changes relative to the control animals Data are presented as means ± SEM. Significant difference between PEDV JS-A and control group are expressed with their *p*. ^*^p < 0.05; ^**^p < 0.01.

At 3 dpi, PEDV JS-A infection downregulated TLR3, TLR4, TLR7, TLR8, and TLR9 expression, as compared to the control group. Further, the corresponding downstream molecules TRIF, MyD88 (subunits A and B), and TRAF6 were significantly downregulated, eventually leading to down-regulation of the NF-κB pathway (*p* < 0.05) (Figures 6A,B). However, at 21 dpi, TLR4, TLR7, and corresponding downstream MyD88 (subunits A and B) were up-regulated, further up-regulating NF-κB. We found that FcRn expression was down-regulated at 4 dpi, yet up-regulated at 21 dpi, and the regulation of FcRn was closely related to regulation of NF-κB (Figures 6C,D).

## Discussion

Despite the use of commercially inactivated vaccines, the characteristics of PED infection and its epidemiology are highly significant, with morbidity and mortality rates approaching 100% in piglets (Pan et al., 2012). In 2013, a sudden PED outbreak occurred in the United States and spread quickly throughout the country, as well as Canada and Mexico, resulting in high mortality of newborn piglets and serious financial problems (Mole, 2013; Stevenson et al., 2013; Vlasova et al., 2014).

In this study, the isolated JS-A strain was more similar to the PEDV GD-1 strain, which was distant from classical strains such as CV777, LZC, and SM98, suggesting that PEDV is continuously evolving, with variation in the epidemiological process. Therefore, vaccines made from classical strains cannot provide effective protection, which is consistent with current findings. The PEDV S gene evolutionary tree and homology were further analyzed. The phylogenetic tree was divided into two evolutionary branches, namely G1 and G2 groups. The G1 group contained classic strains such as CV777, DR13, and SM98. The G2 group has been the most prevalent strain since 2010. JS-A, which has been separated this time, also belongs to G2a. This shows that JS-A is indeed the dominant strain in current pig farms. Induced stronger primary and anamnestic immune responses. The JS-A strain belongs to the non-S-INDEL, which is highly pathogenic.

To study the pathogenicity of PEDV JS-A, 5-day-old piglets were inoculated orally. As expected, vomiting and severe diarrhea were observed in piglets from 1 to 6 dpi, indicating that JS-A was pathogenic to newborn piglets. Fecal swabs were collected from piglets infected with PEDV, and viral fecal shedding was detected by real-time PCR. PEDV RNA was detected from 1 to 14 dpi, suggesting that PEDV infection *via* fecal-oral contamination may be the main transmission route of piglet diarrhea in pig farms. Previously, it has been reported that coronavirus HKU15 may be transmitted *via* the respiratory route, in addition to fecal–oral transmission (Woo et al., 2017; Li et al., 2018). Notably, we only observed microlesions in the jejunum and ileum of piglets infected with JS-A, but PEDV infection did not cause death of newborn piglets, despite drowsiness, anorexia, and watery diarrhea in these animals. These results suggest that PEDV JS-A has a milder toxicity and may be a weak strain. At the same time, there were slight differences in body weight and no significant differences in body temperature during infection.

We also tested the serum-specific IgG, IgA, and neutralizing antibody levels in piglets at 7, 14, and 21 dpi and found that the antibody levels of IgG and IgA gradually increased with time. Further, the serum IgA level was higher than that of IgG. PEDV-challenged protection during the first infection was positively correlated with IgA and IgG antibodies in intestinal-associated lymphoid tissues and blood (de Arriba et al., 2002), indicating that VN antibody can be detected in piglets inoculated with PEDV JS-A, and the titer of VN antibody gradually increases and remains high at the end of the experiment (Chen et al., 2016). In our study, PEDV VN antibody was detected as early as 7 dpi in piglets infected with PEDV. The antibody titer of VN increased and remained high up to 21 dpi, at which point all pigs in the challenged group recovered completely.

*In vitro* studies have proven to be highly dependent on strains, and cell types cannot be used to assess innate immune responses against low virulence strains (Kint et al., 2015). PEDV has been reported to induce innate immune responses *in vivo*. However, few studies have been reported. Therefore, in this study, *in vivo* models were used to further observe the natural TLRs and signaling pathway homeostasis regulated by the gut microbiota. Natural immunity is the first line of host defense against a variety of pathogenic infections (Li et al., 2013). Furthermore, *in vitro* PEDV N and E protein mediated NF-κB activation and had effects on cell growth and ER stress, upregulating IL-8 expression (Xu et al., 2013; Cao et al., 2015b). However, PEDV non-S-INDEL N and nsp1 protein inhibited NF-κB activation (Ding et al., 2014; Zhang et al., 2017). MyD88 is an essential component of the innate immune response to SARS-CoV infection in mice *in vivo* (Totura et al., 2015). The pathogenesis of PEDV is strain specific. The lack of TRAF6 leads to enhanced viral replication and a significant reduction in the production of type I IFN after infection with RNA virus. *In vivo*, PEDV non-S-INDEL down-regulates the NF-κB signaling pathway through negative regulation of TLR4, TLR7, TLR8, and TLR9, resulting in the final attenuation of MyD88, TRIF, and TRAF6 gene expression (Temeeyasen et al., 2018).

The IgA plays a major role in the mucosal anti-PEDV infection immunity. However, recent studies have found that IgG also plays an important role against pathogen infection in mucosal sites, while the neonatal Fc receptor (FcRn) is the only IgG transport receptor (Tsuruta et al., 2012). Down-regulation of FcRn and pIgR has been shown in the tracheal mucosa of SHIV/SIV-infected rhesus macaques (Wang and Yang, 2016; Li et al., 2017). Further, transmissible gastroenteritis virus infection up-regulates FcRn through TLR3 and RIG-I in porcine intestinal epithelial cells (unpublished results). PEDV JS-A can down-regulate NF-κB signaling pathway by inhibiting mRNA expression of TLR3, TLR4, TLR7, TLR8, and TLR9 at 4 dpi, resulting in the final attenuation of FcRn gene expression. In contrast, FcRn expression was up-regulated through TLR3, TLR4, TLR8 at 21 dpi.

In conclusion, we successfully isolated the PEDV JS-A strain, and phylogenetic analysis indicated that the major parent of JS-A strain was identified as strain FL2013 or GD-1. The study results showed that JS-A was a variant PEDV strain with weak pathology to piglets, compared to other emerging strains. High levels of serum IgG, IgA, and VN antibodies were also detected in the sera of infected piglets at 21 dpi. In addition, inoculation of piglets with JS-A suppressed FcRn expression *via* TLR, RIG-I, and NF-κB mRNA expressions at 4 dpi, yet induced FcRn expression at 21 dpi in infected intestinal mucosa. All results showed that innate immunity was suppressed at 4 dpi and that innate immunity might have been activated by the virus at 21 dpi. The down-regulated expression of FcRn resulted in its incapacity to transport antibodies, providing another insight into the immune escape strategy of PEDV.

## Data Availability Statement

The datasets generated for this study can be found in the PEDV JS-A (GenBank: MH748550.1).

## Ethics Statement

The animal study was reviewed and approved by the Scientific Ethic Committee of Huazhong Agricultural University; Huazhong Agricultural University. Written informed consent was obtained from the owners for the participation of their animals in this study.

## Author Contributions

SQ and ZL wrote the paper and conceived and initiated the study. SQ, WZ, and XJ extracted the data set. SQ, WZ, XJ, ZS, YZ, and YX performed the analysis. All authors reviewed the manuscript.

### Conflict of Interest

The authors declare that the research was conducted in the absence of any commercial or financial relationships that could be construed as a potential conflict of interest.
